# Opposite effects of high- and low-frequency transcranial random noise stimulation probed with visual motion adaptation

**DOI:** 10.1038/srep38919

**Published:** 2016-12-09

**Authors:** Gianluca Campana, Rebecca Camilleri, Beatrice Moret, Filippo Ghin, Andrea Pavan

**Affiliations:** 1 Dipartimento di Psicologia Generale, University of Padova, Via Venezia 8, 35131 Padova, Italy; 2 Human Inspired Technology Research Centre, University of Padova, Via Luzzati 4, 35122 Padova, Italy; 3 School of Psychology, University of Lincoln, Brayford Pool, Lincoln LN6 7TS, UK

## Abstract

Transcranial random noise stimulation (tRNS) is a recent neuro-modulation technique whose effects at both behavioural and neural level are still debated. Here we employed the well-known phenomenon of motion after-effect (MAE) in order to investigate the effects of high- vs. low-frequency tRNS on motion adaptation and recovery. Participants were asked to estimate the MAE duration following prolonged adaptation (20 s) to a complex moving pattern, while being stimulated with either sham or tRNS across different blocks. Different groups were administered with either high- or low-frequency tRNS. Stimulation sites were either bilateral human MT complex (hMT^**+**^) or frontal areas. The results showed that, whereas no effects on MAE duration were induced by stimulating frontal areas, when applied to the bilateral hMT^**+**^, high-frequency tRNS caused a significant decrease in MAE duration whereas low-frequency tRNS caused a significant corresponding increase in MAE duration. These findings indicate that high- and low-frequency tRNS have opposed effects on the adaptation-dependent unbalance between neurons tuned to opposite motion directions, and thus on neuronal excitability.

In the last 15 years, there has been an exponential growth in the use of transcranial electrical stimulation (tES) for modulating neuronal excitability and for the induction and investigation of neuroplasticity effects. A recently developed brain stimulation technique is the transcranial random noise stimulation (tRNS), consisting of the application of alternating current over the cortex at random frequencies. The majority of studies investigated the effects of tRNS on cortical excitability by targeting the motor cortex and measuring TMS-induced motor-evoked potentials (MEP). Despite an increase of cortical excitability has been found by applying tRNS with a wide spectrum of frequencies (0.1–640 Hz)^1^,^2^ a number of studies investigated the effect of low- (lf-tRNS; 0.1–100 Hz) vs. high-frequency tRNS (hf-tRNS; 100–640 Hz). There is electrophysiological evidence that hf-tRNS increases cortical excitability for 40 minutes to 1 hour post-stimulation^1^,^3^, at least for intensities equal or larger than 1 mA^2^ and when duration of stimulation was equal or longer than 10 minutes^4^. Besides, hf-tRNS over motor areas enhances motor learning^5^ and reduces task-related activity in frontal regions^6^,^7^. On the other hand, lf-tRNS was found to have no measurable effects on excitability of the motor cortex^1^.

At a perceptual level, most studies investigated the effect of tRNS on neuroplasticity with perceptual learning paradigms: visual perceptual learning was found to be enhanced when concurrent hf-tRNS was applied over early visual areas^8^,^9^,^10^,^11^,^12^,^13^, whereas lf-tRNS produced a moderate enhancement^11^. Hf-tRNS was found to increase excitability in the auditory cortex, as measured with EEG auditory resting state responses^14^, and both hf-tRNS and lf-tRNS (but not full-spectrum tRNS) were able to reduce tinnitus loudness and distress^15^,^16^. Thus, whereas hf-tRNS seems to generally enhance cortical excitability and neuroplasticity, the effects of lf-tRNS are less evident.

In the present study, we employed the well-known phenomenon of motion aftereffect (MAE) in order to investigate the effects of hf-tRNS vs. lf-tRNS on motion adaptation and recovery from adaptation. MAE is a phenomenon in which a stationary (or flickering) stimulus appears to move in the opposite direction with respect to a previously observed moving pattern^17^. Adaptation of neural populations tuned to specific motion directions, and the resulting imbalance in the activity between units responding to opposite directions has been proposed as the cause of this perceptual illusion^17^,^18^,^19^,^20^. Adaptation is considered a form of gain control that increases the efficiency of stimulus encoding, and it takes place at multiple levels of visual motion processing^17^,^21^,^22^. Visual area V5/MT seems to play an important role in visual motion processing and in the generation of the MAE, in particular when complex moving patterns are used^23^,^24^.

Different studies investigated the areas involved in MAE. Single cell recordings in the middle temporal (MT) visual cortex of owl monkeys show that following adaptation to a moving pattern, cells tuned to the opposite direction display increased responsiveness while neurons tuned to the adapting direction were depressed^25^,^26^,^27^. In humans, localization of the neural substrate of MAEs within the hMT^+^ is supported by functional magnetic resonance imaging (fMRI) studies^24^,^28^,^29^,^30^,^31^,^32^,^33^. For example, Hogendoorn and Verstraten^33^ using fMRI, showed that the perceived MAE direction is not encoded in the same way as real motion. Rather than producing an imbalance between opponent pairs of motion detectors, suppression of motion detectors during adaptation resulted in a shift of the population response (also involving detectors tuned to the orthogonal directions) of motion sensitive neurons in hMT^+^. Though motion selectivity was observed in the striate and extrastriate areas (i.e., V1, V2, V3, V4), this modulation of the population response was only observed in area hMT^+^.

Transcranial magnetic stimulation (TMS) studies found that an impairment of the functional integrity of area V5/MT (but not of area V1), during the retention period, decreased MAE duration^23^,^34^, and a similar finding was obtained with tDCS over the same areas^35^. Other neuroimaging and TMS studies found the involvement of earlier visual areas, but in these studies simple translational motion instead of complex motion patterns were used^28^,^29^,^36^,^37^. In fact, the processing of complex motion is mainly mediated by the area hMT^+^^38^,^39^, and the MAE produced by adaptation to a complex moving pattern is likely to be subserved by the same regions^23^.

In order to understand the neural effects of lf- and hf-tRNS on visual areas, we employed the MAE resulting from adaptation to a complex moving pattern, and measured the MAE duration while participants were administered either Sham stimulation, lf-tRNS or hf-tRNS. The spatial specificity of tRNS was investigated by testing the effect of tRNS over bilateral hMT^+^ vs. frontal sites not involved in motion processing. In a previous study where tDCS was used over the left V5/MT area, a decrease of MAE duration was observed with both anodal and cathodal stimulation^35^. In the present study we assess whether lf- and hf-tRNS produce the same or different effects on MAE duration, and whether the effect consists of either a decrease (as found with both tDCS and TMS) or an increase in MAE duration.

## Methods

### Participants

Two different groups of twelve participants took part in the main Experiment and a different group of twelve participants took part in two subsequent control Experiments. All participants had normal or corrected-to-normal vision and they were unaware of the purpose of the study.

All participants were screened by means of a structured interview for any condition that may increase the risks associated with the use of transcranial electrical stimulation. All methods, including transcranial electrical stimulation, were performed in accordance with the relevant guidelines and regulations^40^,^41^. In addition, all participants gave written informed consent according to the Declaration of Helsinki. The study (consisting of three experiments) was approved by the Local Ethics Committee at the University of Padova, where the data were collected.

### Apparatus

Stimuli were generated using Matlab and Psychtoolbox^42^,^43^ and displayed on a 22-inch Philips Brilliance 202P4 monitor with a refresh rate of 60 Hz and a resolution of 1280 × 1024 pixels. The monitor was luminance-calibrated (gamma-corrected with γ = 1). Participants sat in a dark room at a viewing distance from the monitor of 57 cm. Each pixel subtended ~1.7′ (0.028 deg). Viewing was binocular. They were instructed to fixate the centre of the screen and underwent practice blocks to familiarize them with the stimuli and task.

### Stimuli and task

Adapting stimuli consisted of a checkerboard pattern composed of a radial grating rotating clockwise or counter-clockwise (16 cycles, 2.5 Hz, 0.49 Michelson contrast) superimposed on a concentric grating expanding or contracting at 2.5 Hz. The concentric grating had 4 cycles and a contrast of 0.5. The resulting contrast was 0.98 (Michelson contrast). Adapting and test patterns had the same contrast. The resulting temporal frequency was 5 Hz. Adapting stimuli were chosen on the basis of previous brain stimulation experiments with repetitive transcranial magnetic stimulation^23^, and have been demonstrated to produce strong and reliable MAE^30^.

**Figure 1:**
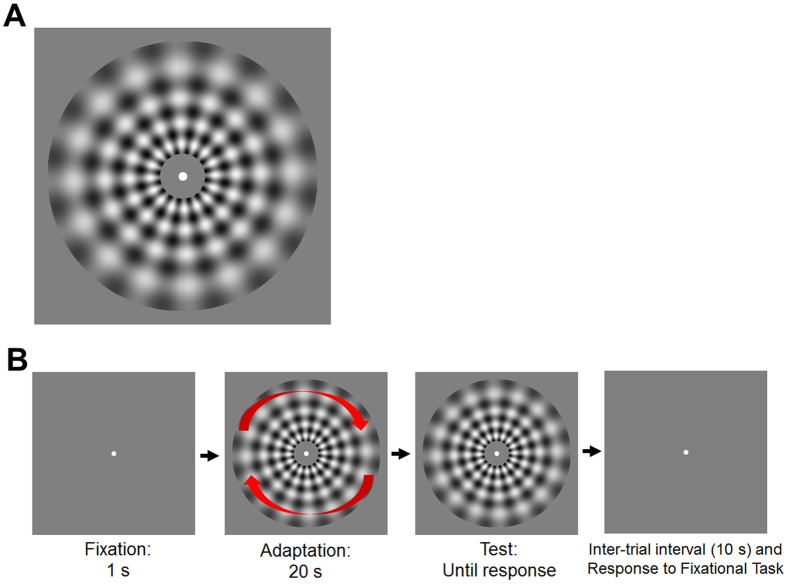
(**A**) An example of the checkerboard pattern used in the Experiments. During the adaption phase, the grating was rotating and contracting or expanding, whereas during the test phase it was stationary. (**B**) A schematic illustration of the timeline of a trial.

Stimuli were viewed throughout a circular annulus with an outer radius of 5.5 deg and an inner radius of 1 deg. A white fixation point (diameter 0.38 deg) was placed at the center of the stimuli (Fig. 1A). The adapting pattern was presented at the center of the screen and observers had to maintain their fixation on the white fixation point. The adapting stimulus was presented for 20 s. After the adaptation period, we presented a stationary version of the adapting pattern (test stimulus) and observers judged both the direction of the illusory motion and when it stopped by pressing one of two designated keys on a standard Italian keyboard. In particular, observers had to press the “Right Arrow” key when the illusory clockwise motion stopped and the “Left Arrow” key when the illusory counter-clockwise motion stopped.

The motion direction of the adapting pattern was randomized on a trial basis with the constraint that the same adapting direction could not be repeated for more than three consecutive trials. Observers were adapted to two clockwise directions: clockwise outward (superimposing a clockwise radial pattern and an expanding circular pattern) and clockwise inward (superimposing a clockwise radial pattern and a contracting circular pattern), and to two counter-clockwise directions: counter-clockwise outward (superimposing a counter-clockwise radial pattern and an expanding circular pattern) and counter-clockwise inward (superimposing a counter-clockwise radial pattern and a contracting circular pattern).

During the adapting phase of each trial, observers carried out a secondary task at fixation. For this secondary task, a similar procedure was used to that reported by Hogendoorn and Verstraten^33^. During adaptation, the size of the central fixation point became smaller (from 0.38 deg to 0.09 deg) for just one frame (~17 ms); the task of the observer was to detect and count these changes during the adapting phase of each trial, which occurred between one and four times. During the inter-trial interval, observers verbally reported the number of fixational changes. The purpose of this secondary task was to ensure that participants maintained central fixation and kept their attention engaged^28^,^32^,^33^. We did not provide feedback on this secondary task. To allow recovery from adaptation, the inter-trial interval was 10 s (Fig. 1B). There were 24 trials for each block (i.e., 6 trials per each adapting direction).

### Procedure

In the main Experiment, twelve participants underwent bilateral hf-tRNS of the area hMT^+^ and the remaining twelve participants underwent bilateral lf-tRNS of the same area. Within a session, 2 blocks of 24 trials each (with a pause between the first and the second block) were administered: the first one with Sham stimulation, the second one with tRNS. The stimulation was delivered throughout trials, therefore including adaptation, test and MAE duration judgments^35^.

The order of Sham vs. tRNS stimulation was not counterbalanced across participants; administering the real brain stimulation on the first block would have modulated the cortical excitability, thus affecting the second block with Sham stimulation, making impossible to distinguish between the effects of real vs. sham stimulation. In order to control for this, we performed an additional experiment (control Experiment 1), in which participants underwent Sham stimulation for two consecutive blocks with electrodes positioned bilaterally over the area hMT^+^. Two blocks of 24 trials each (with a pause between the first and the second block) were administered. Additionally, we devised a second control experiment (control Experiment 2) in order to test whether the modulation of MAE duration due to tRNS was specific for the bilateral stimulation of the area hMT^+^. In the latter control experiment hf-tRNS was applied over the frontal areas.

### Transcranial electrical stimulation

Electrical stimulation was delivered using a battery-driven stimulator (BrainSTIM, EMS) through a pair of saline-soaked sponge electrodes. Impedance was always kept below 5 Kohms. The tRNS consisted of an alternating current (1.5 mA intensity with no offset) applied at random frequencies ranging from 100 to 640 Hz for the hf-tRNS and from 0.1 to 100 Hz for the lf-tRNS. The stimulation started ~60 s before the beginning of the second block and lasted for the whole duration of the block (approximately 17–18 min). Current intensity was linearly increased up to 1.5 mA during the first 30 s and was then kept constant until the end of the block. Electrodes had an area of 25 cm^2^. For the bilateral stimulation of the area hMT^+^, the two electrodes were placed at a site located ~3 cm above the inion and ~5 cm anteriorly on the left and on the right, respectively. In this way, in both conditions we were able to stimulate the targeted areas of both hemispheres. Sham stimulation was delivered by linearly increasing current intensity for 30 s up to 1.5 mA, and decreasing it during the successive 30 s up to 0 mA, just before the beginning of a block. The electrodes were kept in place with bandages. Electrode montage was performed before the beginning of the first block and kept unaltered until the end of the second block.

## Results

### Main Experiment

**Figure 2:**
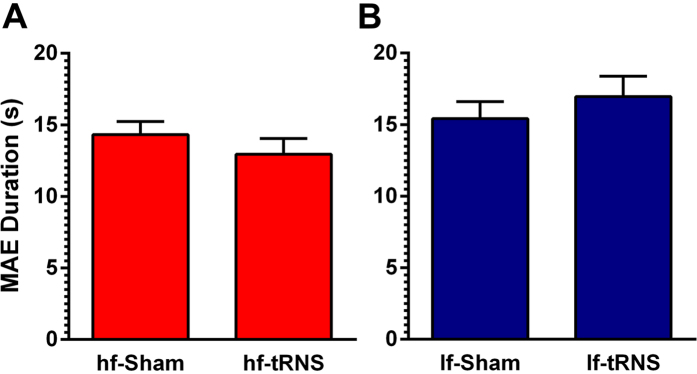
(**A**) Mean MAE duration (in seconds) is shown, separately for Sham and hf-tRNS and (**B**) for Sham and lf-tRNS. Stimulation was delivered over the area hMT^+^. Error bars ± SEM.

Figure 2 displays the results of the main Experiment. The mean MAE duration is shown as a function of the stimulation condition. A Shapiro-Wilk test of normality did not yield any significant result for any level of the considered variables. A mixed ANOVA including stimulation effectiveness (Sham vs. tRNS) as within-subjects factor and stimulation type (hf- vs. lf-tRNS) as between-subjects factor, reported no effect of stimulation effectiveness (*F*_1,22_ = 0.63, *p* = 0.80, *η*_*p*_^*2*^ = 0.003) and no effect of the stimulation type (*F*_1,22_ = 2.28, *p* = 0.14, *η*_*p*_^*2*^ = 0.09).

However, a significant interaction between the two factors (*F*_1,22_ = 17.34, *p* = 0.001, *η*_*p*_^*2*^ = 0.44) suggests that tRNS may have different effects depending on the type of stimulation administered. Post-hoc paired t-tests confirmed this hypothesis: whereas hf-tRNS significantly decreased MAE duration (1.34 s [SEM: 0.42 s] shorter) with respect to Sham stimulation (*t*_11_ = 3.14, *p* = 0.009, *r* = 0.69), lf-tRNS significantly increased MAE duration (*t*_11_ = −2.82, *p* = 0.017, *r* = 0.65) with respect to Sham stimulation by a similar amount (1.54 s [SEM: 0.52 s] longer).

### Control Experiments

**Figure 3:**
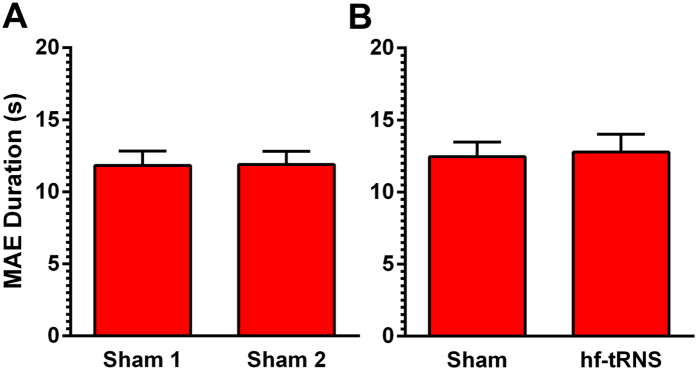
(**A**) Mean MAE duration (in seconds) is shown for two consecutively administered sessions of Sham stimulation over the area hMT^+^. (**B**) Mean MAE duration is shown for Sham and hf-tRNS on frontal areas. Error bars ± SEM.

Figure 3A shows the mean MAE duration estimated in control Experiment 1. A paired-sample t-test did not report any significant difference between the two conditions (*t*_11_ = −0.116, *p* = 0.91, *r* = 0.035). Figure 3B shows the mean MAE duration in control Experiment 2. The t-test did not report any significant differences between the two conditions (*t*_11_ = −0.442, *p* = 0.67, *r* = 0.13).

## Discussion

In this study, we investigated the effects of lf- and hf-tRNS using a standard motion adaptation paradigm. The rationale was to assess the effects of these stimulation regimes over area hMT^+^ during adaptation to complex motion. The results of the main Experiment showed a double dissociation between the effect of hf- and lf-tRNS over bilateral hMT^+^: whereby hf-tRNS decreased MAE duration, and lf-tRNS over the same areas increased the perceived MAE duration by a similar amount. Additionally, two control experiments ruled out the possibility that these results (i) depended on the stimulation order; that is to the fact that in the main experiment, in order to prevent crossover effect of the stimulation, Sham always preceded real stimulation, and (ii) were unspecific with respect to the stimulation site. In fact, modulation of the MAE duration only occurred when lf- and hf-tRNS were delivered over the area hMT^+^, whereas neither lf- nor hf-tRNS delivered over frontal areas were able to modulate MAE duration.

Differently from tDCS, where a decrease of MAE duration was obtained when stimulating V5/MT regardless of the current polarity (i.e., with both anodal and cathodal stimulation^35^), here we show for the first time that the high and low range of frequencies used with tRNS can make a substantial difference, producing opposite effects on the recovery from motion adaptation. One possible explanation for the effect of hf-tRNS is that the application of high-frequency alternating current modulates the activation of the sodium channels^44^. Based on physiological and behavioural evidence, tRNS (considering the whole frequency spectrum) may induce a summation of small depolarizing currents throughout the repetitive activation of the sodium channels and the consequent influx of Na+ ions inside the membrane^11^,^45^. We suggest that hf-tRNS may interfere with the depressed state of the adapted neurons repolarizing the membrane near to its resting state, thus reducing the imbalance between adapted and non-adapted motion sensors and resulting in a shorter MAE duration.

The neural mechanisms underlying the opposite effect of lf-tRNS on MAE duration is less clear. The longer MAE duration induced by the lf-tRNS may result from a stronger induction of the adaptation state specific for motion detectors selective to the adapting direction. There is physiological evidence that alternating current induces different effects depending on specific frequency ranges and leads to different responses between neural subpopulations^46^. Freeman and coworkers^46^ showed that stimulation at 5, 10 and 25 Hz influenced presynaptic retinal neurons producing a strong response only near the soma of ganglion cells, whereas stimulations at 100 Hz elicited higher responses both near the soma and over the distal axons. This suggest that low frequency stimulation, differently to high frequency stimulation, may result in a more “focal” activity^46^.

Motion sensors in area hMT^+^ form neural populations whose direction selectivity depends on the complex and non-linear interactions between neurons tuned to different motion directions^47^,^48^. Motion opponency, i.e., inhibition between neurons tuned to opposite motion direction, is likely to underlie the response of these neural populations^49^,^50^. In these neural populations, adaptation of one neuron can reduce the inhibitory input to other neurons thereby increasing their response. We speculate that modulation differences produced by lf-tRNS might result in a fragmentary interaction among neurons inside the neural population coding for a specific direction. When adaptation occurs, this fragmentary interaction might result in a prolonged adaptation state of the motion detectors. Therefore, it is possible that different patterns of modulation occur between adapted and non-adapted neurons when lf-tRNS is applied. However, further investigation is required to assess the effects of tRNS at different frequency ranges in modulating the activity of visual neurons.

## Additional Information

**How to cite this article**: Campana, G. *et al*. Opposite effects of high- and low-frequency transcranial random noise stimulation probed with visual motion adaptation. *Sci. Rep.* **6**, 38919; doi: 10.1038/srep38919 (2016).

**Publisher's note:** Springer Nature remains neutral with regard to jurisdictional claims in published maps and institutional affiliations.
